# Supplementary material for: Coupling between intra- and intermolecular motions in liquid water revealed by two-dimensional terahertz-infrared-visible spectroscopy
Source: Nat Commun. 2018 Feb 28;9:885. doi: 10.1038/s41467-018-03303-y (PMC5830436; doi:10.1038/s41467-018-03303-y)
Supplement: Supplementary file 1 — Supplementary Information [file 41467_2018_3303_MOESM1_ESM.pdf]

Coupling between intra- and intermolecular motions in liquid water revealed by two-dimensional terahertz-infrared-visible spectroscopy

Maksim Grechko, Taisuke Hasegawa, Francesco D'Angelo, Hironobu Ito, Dmitry Turchinovich, Yuki Nagata and Mischa Bonn

Department of Molecular Spectroscopy, Max Planck Institute for Polymer Research, Ackermannweg 10, D-55128 Mainz, Germany

Supplementary Information

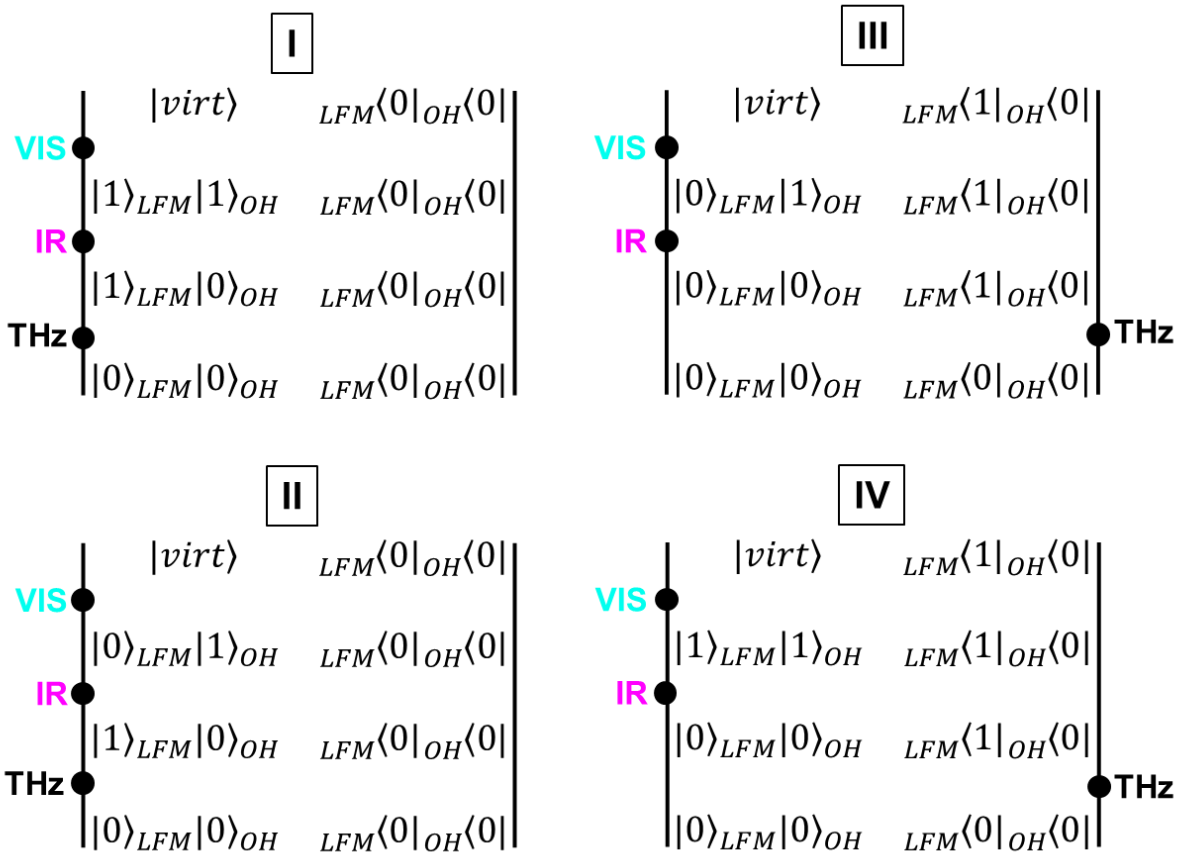

Supplementary Figure 1. Feynman diagrams representing the possible Liouville excitation pathways in the 2D TIRV spectroscopy. The indexes label quantum states of the LFM and O-H stretch oscillators, respectively. $|virt>$ is the virtual state. Black circles represent interaction with the corresponding laser fields. The diagrams I and II (III and IV) correspond to the $\omega_{\mathrm{VIS}} + \omega_{\mathrm{IR}} + \omega_{\mathrm{THz}}$ ($\omega_{\mathrm{VIS}} + \omega_{\mathrm{IR}} - \omega_{\mathrm{THz}}$) four-wave mixing.

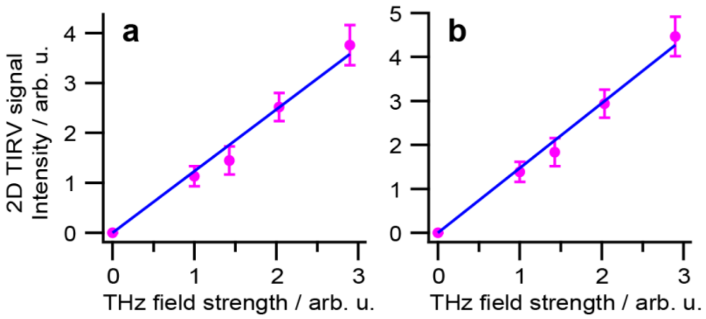

Supplementary Figure 2. Dependence of the 2D TIRV signal intensity on the strength of the THz field for water. Intensity of the 2D TIRV signal (magenta filled circles) is measured for different intensities of the THz pulse for 100% D_2O (**a**) and 100% H_2O (**b**). The blue lines in **a** and **b** show linear fit to the data.

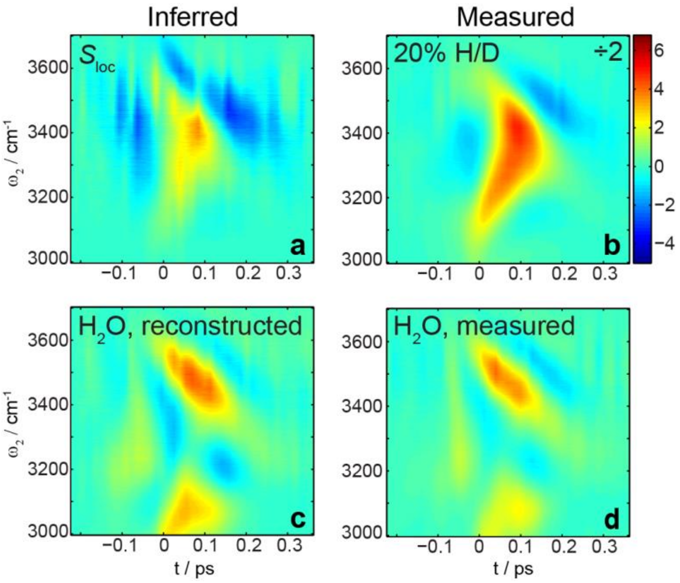

Supplementary Figure 3. Inferred time-domain response for isolated and coupled O-H oscillators. 2D TIRV time-domain data for: **a**, S_{loc} for isolated O-H oscillators derived from the 5% spectrum in Fig. 3g; **c**, reconstructed for 100% H_2O by subtracting the 50% and 20% H/D spectra. For comparison, the 20% and 100% data from Fig. 3 are reproduced in panels **b** and **d**.

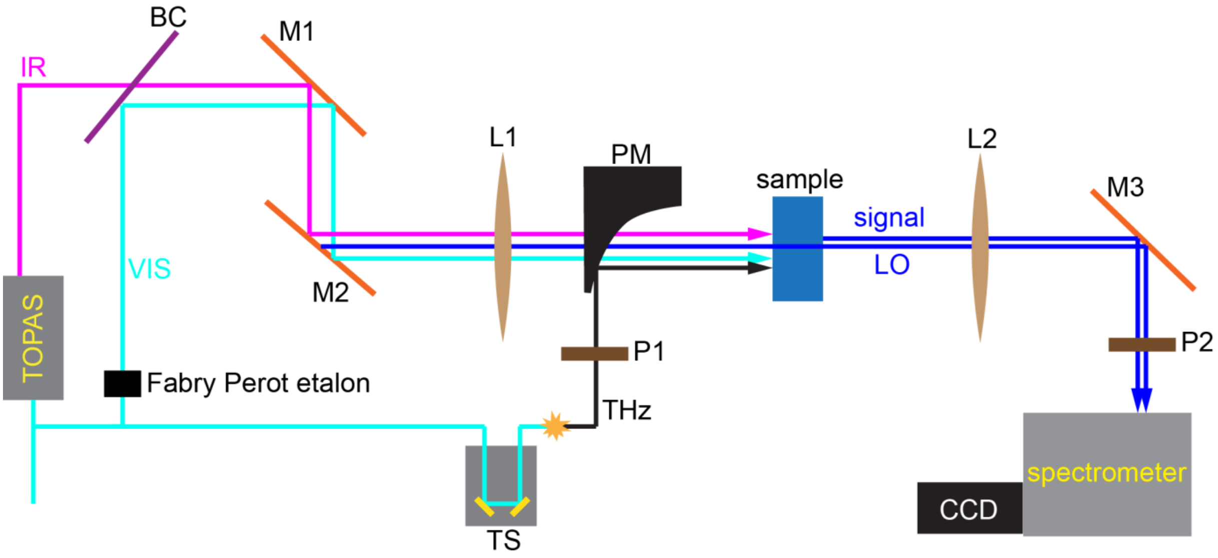

Supplementary Figure 4. Optical layout for the 2D TIRV spectroscopy.

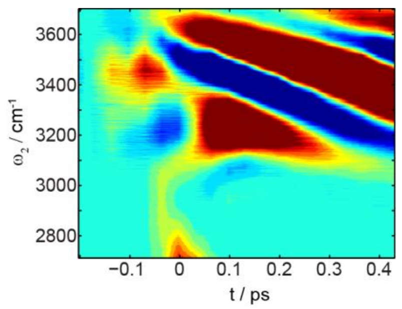

Supplementary Figure 5. Time-domain 2D TIRV data for CaF_2. The data is identical to that in Fig. 2e but axes limits are chosen to show the weak non-resonant signal at $\omega_2 \leq 2800\ \mathrm{cm}^{-1}$.

Supplementary Note 1: Intensity spectrum of the THz laser pulse at the position of the sample

To characterize intensity spectrum of the THz laser pulse at the spatial overlap with the IR and VIS beams we employ terahertz-field-induced second harmonic generation (TFISH) in nitrogen gas. To this end, we block the IR beam and measure the spectrum of the UV light at the frequencies of $2\omega_{\mathrm{VIS}} \pm \omega_{\mathrm{THz}}$ (≈400 nm) produced by four wave mixing of the VIS and THz pulses in nitrogen. Intensity of such signal is given by $\left|\chi^{(3)}\right|^2 I_{\mathrm{VIS}}^2 I_{\mathrm{THz}}$, where $\chi^{(3)}$ is the third-order nonlinear optical susceptibility of nitrogen, I_{VIS} and I_{THz} are intensities of the VIS and IR beams, respectively. Indeed, Supplementary Fig. 6a shows linear dependence of the signal intensity on the intensity of the THz pulse which confirms the nature of the signal. Magenta line in Supplementary Fig. 6b shows the intensity spectrum of the TFISH signal. Comparison of the TFISH spectrum with the second harmonic of the VIS pulse (black line in Supplementary Fig. 6b), which is generated at the mirrors (M1 and M2 in Supplementary Fig.4), demonstrates that the former is produced by $2\omega_{\mathrm{VIS}} - \omega_{\mathrm{THz}}$ wave mixing. Thus, because we use narrowband VIS pulse the magenta trace in Supplementary Fig. 6b amounts to the THz spectrum shifted by $2\omega_{\mathrm{VIS}}$. The THz spectrum derived from the TFISH measurement (Fig. 2b) is in a good agreement with the spectrum measured previously by the air based coherent (ABC) detection (Supplementary Fig. 6c,d)[1]. We note that the absolute-value 2D TIRV spectra are not sensitive to the variation of the phase across the THz spectrum (see Supplementary Note 2). We also stress that for the ABC detection the THz beam is overlapped in space with a sampling beam in the ABC detector. The spatial overlap of the THz and sampling beams in the ABC detector can be different from the overlap of the THz beam with the IR and VIS beams in the 2D TIRV measurement. This difference can be the reason, at least partially, for the difference in the spectra of the THz pulse measured by different methods.

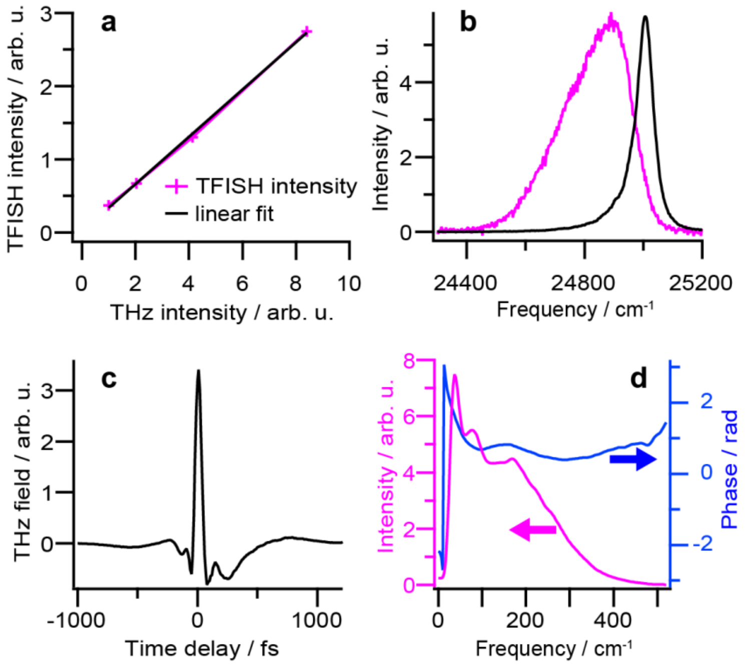

Supplementary Figure 6. Intensity spectrum of the THz pulse. a, Intensity of the TFISH signal for different intensities of the THz laser pulse. The magenta trace shows experimental data and the black line shows a linear fit. **b**, Spectrum of the TFISH signal (magenta) and of the second harmonic of the VIS pulse (black). **c**, THz pulse measured using the air based coherent detection. **d**, Intensity spectrum (magenta) and phase (blue) of the THz pulse obtained by Fourier transform of the signal in **c.**

Supplementary Note 2: Perturbation formalism for the 2D TIRV spectroscopy

In this section, we derive the relationship between the signal measured by the 2D TIRV spectroscopy and the third-order nonlinear response function $S^{(3)}$ of a sample. Electric field $E^{(3)}$ of the 2D TIRV signal emitted by a sample after interactions with the THz, IR and VIS pulses is proportional to the third-order nonlinear polarization generated by these interactions. Thus, by employing the frequency domain formalism (Eq. (5.32) in Ref.[2]) we obtain for time delay τ of the THz pulse:

$$E^{(3)}(t,\tau) \propto \iiint d\omega_1 d\omega_2 d\omega_3 S^{(3)}(\omega_1+\omega_2+\omega_3,\omega_1+\omega_2,\omega_1)\, E_{\mathrm{THz}}(\omega_1)e^{-i\omega_1\tau}E_{\mathrm{IR}}(\omega_2)E_{\mathrm{VIS}}(\omega_3)e^{-i\omega_s t}, \qquad (1)$$

where frequency $\omega_\mathrm{s}=\omega_1+\omega_2+\omega_3$; $E_{\mathrm{THz}}(\omega_1)$, $E_{\mathrm{IR}}(\omega_2)$ and $E_{\mathrm{VIS}}(\omega_3)$ are electric fields of the THz, IR and VIS pulses at the frequencies ω_1, ω_2 and ω_3, respectively. Dispersion of the signal by the grating of the spectrometer is given by Fourier transform of the signal field over time t:

$$E^{(3)}(\Omega_3',\tau) = \mathrm{FT}\left[E^{(3)}(t,\tau)\right] \propto \iiint d\omega_1 d\omega_2 d\omega_3 S^{(3)}(\omega_1+\omega_2+\omega_3,\omega_1+\omega_2,\omega_1)\, E_{\mathrm{THz}}(\omega_1)e^{-i\omega_1\tau}E_{\mathrm{IR}}(\omega_2)E_{\mathrm{VIS}}(\omega_3)\delta(\Omega_3'-\omega_\mathrm{s}), \qquad (2)$$

where δ is the Dirac delta function. Because after the grating positive and negative frequency components of light travel in the same direction the signal A measured by the square-law detector (CCD camera) for frequency $\Omega_3 \geq 0$ in the presence of the local oscillator (LO) and integrated over time period T is given by:

$$A(\Omega_3,\tau) \propto \int_T dt\left[E^{(3)}(\Omega_3,\tau)e^{-i\Omega_3 t}+E^{(3)}(-\Omega_3,\tau)e^{i\Omega_3 t}+E_{\mathrm{LO}}(\Omega_3)e^{-i\Omega_3 t}+E_{\mathrm{LO}}(-\Omega_3)e^{i\Omega_3 t}\right]^2. \qquad (3)$$

where $E_{\mathrm{LO}}(\Omega_3)$ is electric field of the LO at frequency Ω_3. By squaring the term under integral and neglecting highly-oscillating terms we obtain for the heterodyne-detected signal B (interference between the signal field and the LO):

$$B(\Omega_3,\tau) \propto E^{(3)}(\Omega_3,\tau)E_{\mathrm{LO}}(-\Omega_3)+E^{(3)}(-\Omega_3,\tau)E_{\mathrm{LO}}(\Omega_3). \qquad (4)$$

Heterodyne-detected signal depends parametrically on the time delay τ of the THz pulse, which is reflected by the time-domain 2D TIRV data in the experiment. In order to obtain 2D spectra we perform Fourier transform of this signal over τ:

$$Spectrum(\Omega_3,\Omega_1) = \mathrm{FT}[B(\Omega_3,\tau)]$$
$$\propto \mathrm{FT}[E^{(3)}(\Omega_3,\tau)]E_{\mathrm{LO}}(-\Omega_3) + \mathrm{FT}[E^{(3)}(-\Omega_3,\tau)]E_{\mathrm{LO}}(\Omega_3)$$
$$= E^{(3)}(\Omega_3,\Omega_1)E_{\mathrm{LO}}(-\Omega_3) + E^{(3)}(-\Omega_3,\Omega_1)E_{\mathrm{LO}}(\Omega_3). \qquad (5)$$

Using Supplementary Eq. (2) we obtain for $E^{(3)}(\Omega_3,\Omega_1)$:

$$E^{(3)}(\Omega_3,\Omega_1) = \mathrm{FT}[E^{(3)}(\Omega_3,\tau)]$$
$$\propto \iiint d\omega_1 d\omega_2 d\omega_3 S^{(3)}(\omega_1+\omega_2+\omega_3,\omega_1+\omega_2,\omega_1)\, E_{\mathrm{THz}}(\omega_1)\delta(\Omega_1$$
$$-\omega_1)E_{\mathrm{IR}}(\omega_2)E_{\mathrm{VIS}}(\omega_3)\delta(\Omega_3-\omega_{\mathrm{s}}). \qquad (6)$$

In the experiment, we use narrowband VIS pulse for which we approximate the spectrum by delta function at frequency $\Omega_{\mathrm{VIS}} > 0$:

$$E_{\mathrm{VIS}}(\omega_3) = \delta(\Omega_{\mathrm{VIS}}-\omega_3) + \delta(\Omega_{\mathrm{VIS}}+\omega_3). \qquad (7)$$

By substituting Supplementary Eq. (7) into Supplementary Eq. (6) and integrating we obtain:

$$E^{(3)}(\Omega_3,\Omega_1) \propto S^{(3)}(\Omega_3,\Omega_3-\Omega_{\mathrm{VIS}},\Omega_1)E_{\mathrm{THz}}(\Omega_1)E_{\mathrm{IR}}(\Omega_3-\Omega_{\mathrm{VIS}}-\Omega_1)$$
$$+ S^{(3)}(\Omega_3,\Omega_3+\Omega_{\mathrm{VIS}},\Omega_1)E_{\mathrm{THz}}(\Omega_1)E_{\mathrm{IR}}(\Omega_3+\Omega_{\mathrm{VIS}}-\Omega_1). \qquad (8)$$

Because in our experiment $\Omega_1 \ll \Omega_3 + \Omega_{\mathrm{VIS}}$ the frequency $\Omega_3 + \Omega_{\mathrm{VIS}} - \Omega_1$ is beyond the bandwidth of the IR pulse. Thus, $E_{\mathrm{IR}}(\Omega_3+\Omega_{\mathrm{VIS}}-\Omega_1) = 0$ and the second term in Supplementary Eq. (8) vanishes. Similar, for $E^{(3)}(-\Omega_3,\Omega_1)$ we obtain:

$$E^{(3)}(-\Omega_3,\Omega_1) \propto S^{(3)}(-\Omega_3,-\Omega_3+\Omega_{\mathrm{VIS}},\Omega_1)E_{\mathrm{THz}}(\Omega_1)E_{\mathrm{IR}}(-\Omega_3+\Omega_{\mathrm{VIS}}-\Omega_1). \qquad (9)$$

By substituting Supplementary Eqs. (9) and (8) into Supplementary Eq. (5) we obtain for a measured 2D TIRV spectrum:

$$Spectrum(\Omega_3,\Omega_1)$$
$$\propto S^{(3)}(\Omega_3,\Omega_3-\Omega_{\mathrm{VIS}},\Omega_1)E_{\mathrm{THz}}(\Omega_1)E_{\mathrm{IR}}(\Omega_3-\Omega_{\mathrm{VIS}}-\Omega_1)E_{\mathrm{LO}}(-\Omega_3)$$
$$+ S^{(3)}(-\Omega_3,-\Omega_3+\Omega_{\mathrm{VIS}},\Omega_1)E_{\mathrm{THz}}(\Omega_1)E_{\mathrm{IR}}(-\Omega_3+\Omega_{\mathrm{VIS}}-\Omega_1)E_{\mathrm{LO}}(\Omega_3). \ (10)$$

We introduce frequency $\Omega_2 = \Omega_3 - \Omega_{\mathrm{VIS}}$ to write Supplementary Eq. (10) in the alternative form:

$$Spectrum(\Omega_2+\Omega_{\mathrm{VIS}},\Omega_1)$$
$$\propto S^{(3)}(\Omega_2+\Omega_{\mathrm{VIS}},\Omega_2,\Omega_1)E_{\mathrm{THz}}(\Omega_1)E_{\mathrm{IR}}(\Omega_2-\Omega_1)E_{\mathrm{LO}}(-\Omega_2-\Omega_{\mathrm{VIS}})$$
$$+ S^{(3)}(-\Omega_2-\Omega_{\mathrm{VIS}},-\Omega_2,\Omega_1)E_{\mathrm{THz}}(\Omega_1)E_{\mathrm{IR}}(-\Omega_2-\Omega_1)E_{\mathrm{LO}}(\Omega_2+\Omega_{\mathrm{VIS}}). \ (11)$$

Thus, in the experiment, the 2D TIRV spectrum is given by the product of the spectrum of the third-order nonlinear response function with the spectra of the laser pulses. The presence of coupled vibrational resonances at frequencies Ω_1 (THz

frequency range) and Ω_2 (IR frequency range) results in the peaks in the spectrum of the $S^{(3)}$ response function and, therefore, in the 2D TIRV spectrum.

Supplementary Equation (11) shows that 2D TIRV spectra in our experiment are produced by sum of the $S^{(3)}$ spectra in two quadrants, (Ω_2, Ω_1) and $(-\Omega_2, \Omega_1)$. This is due to the utilization of the spectrometer to measure the second frequency axis and is analogous to the summation of the rephasing and non-rephasing spectra in the 2D IR spectroscopy when a spectrometer and the pump-probe geometry are used.

Supplementary Note 3: Change of the 2D TIRV signal intensity with sample composition

The gain of the signal field dE_{sig} produced by FWM of the THz, IR and VIS beams over the distance dx in the sample is given by[3]:

$$dE_{\mathrm{sig}} \propto \chi^{(3)} E_{\mathrm{THz}}(x) E_{\mathrm{IR}}(x) E_{\mathrm{VIS}}(x) e^{i\Delta k x} dx, \qquad (12)$$

where $\chi^{(3)}$ is the third-order nonlinear optical susceptibility of the medium; $E_{\mathrm{THz}}(x)$, $E_{\mathrm{IR}}(x)$ and $E_{\mathrm{VIS}}(x)$ are electric fields of the THz, IR and VIS pulses at the position x inside the sample; $\Delta k = k_{\mathrm{THz}} + k_{\mathrm{IR}} + k_{\mathrm{VIS}} - k_{\mathrm{sig}}$ is the mismatch between the wavevector of the signal and those of the incident waves. Upon propagation into the sample the laser beams are depleted by absorption as well as conversion into the signal field. We neglect the latter because FWM in our experiment is much weaker than the linear absorption. Absorption coefficients for H_2O and D_2O at THz frequencies are similar, therefore we assume them to be equal $\varepsilon_{\mathrm{THz}}$. Also, we assume equal absorption coefficients, $\varepsilon_{\mathrm{IR}}^{\mathrm{OH}}$, for O-H oscillators in H_2O and HOD at IR frequencies. Because water is transparent at the wavelength of the VIS beam we assume its intensity to be constant. Thus, using the Beer–Lambert law and amplitudes E_{THz}^0, E_{IR}^0 and E_{VIS}^0 of the laser fields at the front surface of the sample Supplementary Eq. (12) is:

$$dE_{\mathrm{sig}} \propto \chi^{(3)} E_{\mathrm{THz}}^0 E_{\mathrm{IR}}^0 E_{\mathrm{VIS}}^0 \exp\left[\left(-\frac{\varepsilon_{\mathrm{IR}}^{\mathrm{OH}} C_{\mathrm{OH}} + \varepsilon_{\mathrm{IR}}^{\mathrm{OD}} C_{\mathrm{OD}} + \varepsilon_{\mathrm{THz}} C}{2} + i\Delta k\right) x\right] dx, \qquad (13)$$

where C, C_{OH} and C_{OD} and are the concentrations of all molecules, O-H and O-D oscillators, respectively. By integrating the differential Supplementary Eq. (13) we obtain signal emitted by the sample of thickness L:

$$E_{\mathrm{sig}} \propto \frac{\chi^{(3)}}{\alpha/2 + i\Delta k}\left[e^{(-\alpha/2 + i\Delta k)L} - 1\right]. \qquad (14)$$

Where we have introduced the total extinction coefficient $\alpha = \varepsilon_{\mathrm{IR}}^{\mathrm{OH}} C_{\mathrm{OH}} + \varepsilon_{\mathrm{IR}}^{\mathrm{OD}} C_{\mathrm{OD}} + \varepsilon_{\mathrm{THz}} C$. For 1 mm thick samples that we use $\alpha L \gg 1$ for all isotope dilutions and thus the amplitude of the signal:

$$\left|E_{\mathrm{sig}}\right| \propto \frac{\left|\chi^{(3)}\right|}{\sqrt{\alpha^2/4 + \Delta k^2}}. \qquad (15)$$

For H/D mixtures the $\chi^{(3)}$ is composed of non-resonant $\chi_{\mathrm{OD}}^{(3)} \propto C_{\mathrm{OD}} \langle \beta_{\mathrm{OD}}^{(3)} \rangle$ and resonant $\chi_{\mathrm{OH}}^{(3)} \propto C_{\mathrm{OH}} \langle \beta_{\mathrm{OH}}^{(3)} \rangle$ susceptibilities of O-D and O-H oscillators, respectively. Each of these susceptibilities is proportional to the average molecular response functions, $\langle \beta_{\mathrm{OD}}^{(3)} \rangle$ and $\langle \beta_{\mathrm{OH}}^{(3)} \rangle$, and scales linearly with the concentrations of the corresponding species.

We use Supplementary Eq. (15) to examine how intensities of the signals from O-H and O-D vary with the sample composition. To this end, we utilize absorption

coefficients and wavevector mismatch averaged for the THz and IR frequency range of the 2D TIRV spectra. For 5% H/D mixture the denominator in Supplementary Eq. (15) is ≈1.07 times bigger than for 100% D_2O. By taking into account the reduced concentration of the O-D which diminishes the $\chi^{(3)}_{\mathrm{OD}}$ term in the numerator we obtain that intensity of the D_2O signal in Fig. 3g is 0.95/1.07=0.89 of that in Fig. 3f. We use this coefficient to subtract the two spectra in order to eliminate the D_2O signal in the 5% H/D sample and obtain 2D TIRV spectrum for the isolated O-H stretch oscillator (Fig. 4a and Supplementary Fig. 3a).

For the 20% H/D the denominator in Supplementary Eq. (15) is ~1.5 times bigger than for 100% D_2O due to the increased IR absorption by the O-H groups. Taken together with decline of the $\chi^{(3)}_{\mathrm{OD}}$ by 0.8 this isotope dilution decreases the O-D signal by ~1.8. Sharp decrease of the intensity can largely explain the absence of a discernable D_2O signal in Fig. 3h (note also the scaling factor ÷2 for the figure).

Supplementary Methods

Molecular dynamics (MD) simulation Protocols.

MD simulations were performed to calculate the 2D TIRV signals of water. We employed the POLI2VS water potential[4]. We used the cubic unit cells with the length of 12.426 Å and periodic boundary conditions in all directions. The non-electrostatic intermolecular interaction and the electrostatic interactions including quadrupole terms were smoothly cut off into zero from 6.0 Å to 6.1 Å. The other electrostatic interactions were calculated by the standard Ewald sum. The equations of motion were integrated by the r-RESPA algorithm[5]. A 0.5 fs time step was used for integrating the equations of motion for the intermolecular interactions with the 2nd order symplectic integrator, while a 0.25 fs time step was used for the intramolecular bonding interaction with the 6th order symplectic integrator[6]. In the simulation of the neat H_2O, the system consisted of 64 water molecules. In the simulation for the mixture of 5% H_2O and 95 % D_2O, the system consisted of 58 D_2O and 6 HOD molecules. We randomly generated 20 initial structures for both systems. The constant temperature (NVT) MD simulations were performed for 100 ps to equilibrate each system at 300 K, followed by the production runs. The Nose-Hoover chain algorithm was employed in the constant temperature (NVT) MD runs[7]. After the equilibration, the system temperatures were around 300 K in the constant energy (NVE) MD simulations. We employed the equilibrium and non-equilibrium hybrid algorithm to calculate 2D TIRV signals[8,9]. The IR laser pulses with the strengths of 0.1 V/Å were applied to the system for 0.5 fs. Note that the increase in temperature observed after the laser interactions was ~ 0.5 K. We collected total 10^6 non-equilibrium trajectories from the independent 20 equilibrium trajectories for each system. We performed the NEMD and EMD simulations for 250 fs and collected the polarizabilities and dipole moments every 1 fs. The response functions were calculated in the time periods of $0 \le t_1 \le 250$ fs and $0 \le t_2 \le 250$ fs. The NVE condition was used throughout all the production MD runs.

Equilibrium-non-equilibrium hybrid approach.

The time domain quantum response function for the 2D TIRV signal can be expressed as

$$S^{(3)}_{abcd}(t_1,t_2)=\left(\frac{i}{\hbar}\right)^2 \mathrm{tr}\left\{\left[\hat{\Pi}_{ab}(t_2),\hat{M}_c(0)\right]\left[\hat{M}_d(-t_1),\hat{\rho}^{eq}\right]\right\}, \quad (16)$$

where $\hat{\Pi}_{ab}$ is the ab component of the system polarizability operator, $\hat{M}_c$ is the c component of the system dipole moment operator, $\hat{\rho}^{eq}$ is the density matrix in an equilibrated state, $\mathrm{tr}\{\ \}$ denotes the trace, and $[,]$ denotes the quantum commutator. In the classical limit, the quantum commutators can be replaced by the classical Poisson brackets[2,10]. The 2D TIRV response function (Supplementary Eq. (16)) can be recast as

$$S^{(3)}_{abcd}(t_1,t_2)=\iint\left(\left\{\Pi_{ab}(t_2),M_c(0)\right\}_{\mathrm{PB}}\left\{M_d(-t_1),\rho^{eq}(\mathbf{p},\mathbf{q})\right\}_{\mathrm{PB}}\right)d\mathbf{p}d\mathbf{q}\ , \quad (17)$$

where the integrals are performed over all the classical phase space variables, momenta $\mathbf{p}$ and coordinates $\mathbf{q}$. $\{,\}_{\mathrm{PB}}$ represents the classical Poisson bracket. In the equilibrium-non-equilibrium approach, the first Poisson bracket is evaluated by the non-equilibrium MD (NEMD) simulations, while the second one is evaluated by the equilibrium MD (EMD) simulations. By neglecting higher than second order terms with respect to the electrostatic field E_c^{IR} which is applied to the system during the time period of Δt in the NEMD simulation, we have[11];

$$\left\{\Pi_{ab}(t_2),M_c(0)\right\}_{\mathrm{PB}}\simeq\frac{\Pi_{ab}\left(t_2;E_c^{\mathrm{IR}}(t=0)\right)-\Pi_{ab}\left(t_2;-E_c^{\mathrm{IR}}(t=0)\right)}{2\Delta t E_c^{\mathrm{IR}}(t=0)}\ , \quad (18)$$

where $\Pi_{ab}\left(t_2;E_c^{\mathrm{IR}}(t=0)\right)$ is the polarizability of the system at the time of $t=t_2$ after the irradiation of the laser pulse of E_c^{IR} at $t=0$ in the NEMD simulation and $-E_c^{\mathrm{IR}}$ represents the same laser pulse as E_c^{IR} but its sign is inverted. The second Poisson bracket can be written as

$$\left\{M_d(-t),\rho^{eq}(\mathbf{p},\mathbf{q})\right\}_{\mathrm{PB}}=-\beta\frac{dM_d(-t)}{dt}\rho^{eq}(\mathbf{p},\mathbf{q})=\beta\dot{M}_d(-t)\rho^{eq}(\mathbf{p},\mathbf{q})\ , \quad (19)$$

where β is the inverse temperature of the system. Note that the value of $\dot{M}_d(-t)$ is obtained from the EMD simulation. By substituting Supplementary Eq. (18) and (19) into Supplementary Eq. (17), we have[9,12];

$$S_{abcd}^{(3)}(t_1,t_2) \simeq \beta \frac{\left\langle \left\{ \Pi_{ab}\left(t_2; E_c^{\mathrm{IR}}(t=0)\right) - \Pi_{ab}\left(t_2; -E_c^{\mathrm{IR}}(t=0)\right) \right\} \dot{M}_d(-t_1) \right\rangle}{2\Delta t E_c^{\mathrm{IR}}(t=0)} , \tag{20}$$

where $\langle\ \rangle$ denotes the thermal average. The simulation procedure of Supplementary Eq. (20) is schematically illustrated in Supplementary Fig. 7. To carry out the NEMD simulation under the external field, the interaction term between the system dipole and the external IR laser fields were added to the original Hamiltonian described by the POLI2VS model. This interaction term is expressed as:

$$H^{\mathrm{IR}}(t) = -\mathbf{M}\mathbf{E}^{\mathrm{IR}}(t) \tag{21}$$

where $\mathbf{M}$ is the net dipole of the system and $\mathbf{E}^{\mathrm{IR}}(t)$ is the external electrostatic field.

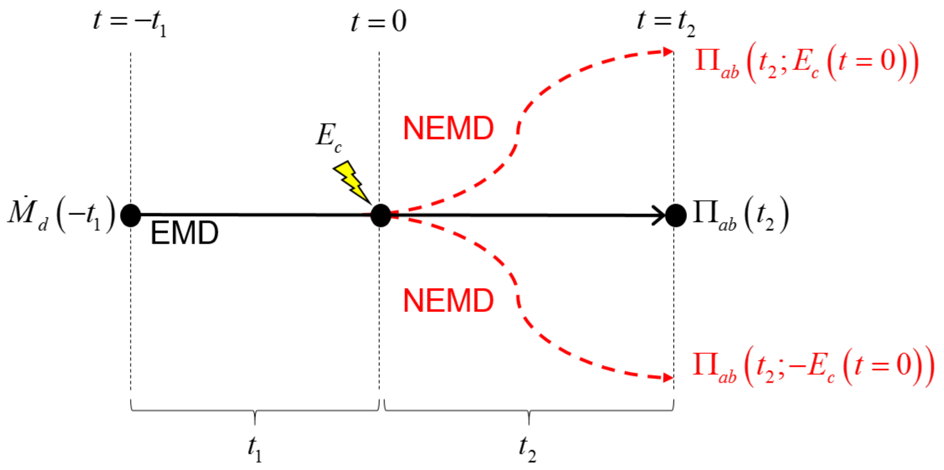

Supplementary Figure 7. The calculation procedure of the time domain 2D TIRV response function by the equilibrium-non-equilibrium approach. The solid line represents the equilibrium trajectory, while the red dashed lines represent the non-equilibrium trajectories which are branched from the equilibrium trajectory at $t=0$ by the irradiation of the IR laser pulses E_c.

Calculation procedure of the 2D TIRV Fourier-transform spectra from simulated time domain signals.

The simulated time domain response functions $S_{abcd}^{(3)}\left(t_1,t_2\right)$ were smoothed by using a window function $f_w(t_1,t_2)=\exp\left[-\left(t_1+t_2\right)^{12}/\tau\right]$, where the parameter $\tau=5\times10^{28}\ \mathrm{fs}^{12}$ was used. Then the smoothed 2D TIRV response function is given by:

$$S_{abcd,w}^{(3)}\left(t_1,t_2\right)=S_{abcd}^{(3)}\left(t_1,t_2\right)f_w\left(t_1,t_2\right) \tag{22}$$

Subsequently, the 2D TIRV spectra in the frequency domain were obtained through the 2D Fourier transformation for the smoothed response function in the time domain (Supplementary Eq. (22)):

$$S_{abcd,w}^{\mathrm{FFT}}(\omega_1,\omega_2)=\sum_{k=0}^{N_1-1}\sum_{l=0}^{N_2-1}\exp\left[-i\omega_1 k\Delta t_1\right]\exp\left[-i\omega_2 l\Delta t_2\right]S_{abcd,w}^{(3)}(k\Delta t_1,l\Delta t_2)\,, \tag{23}$$

where N_1 and N_2 are the numbers of the sampling points along t_1 and t_2 axes, respectively, Δt_1 and Δt_2 are the intervals of the time domain data sets, i is the imaginary unit. With these expressions, the $\left(+\omega_1,+\omega_2\right)$ and $\left(+\omega_1,-\omega_2\right)$ quadrants were calculated for $S_{abcd,w}^{\mathrm{FFT}}(\omega_1,\omega_2)$.

In the experiment, the ω_1 and ω_2 frequencies are measured in time (by scanning the time delay between the THz and IR/VIS pair laser pulses) and frequency (by using a spectrometer) domains, respectively. Because of the frequency domain measurement of the ω_2 the experiment cannot distinguish positive and negative ω_2 frequencies. Thus, in the experiment we measure the sum of the two quadrants $S_{abcd,w}^{\mathrm{FFT}}(+\omega_1,+\omega_2)+S_{abcd,w}^{\mathrm{FFT}}(+\omega_1,-\omega_2)$. Therefore, for the calculated absolute value 2D TIRV spectra in Supplementary Fig. 8 we plot:

$$I_{abcd}(\omega_1,\omega_2)=\left|S_{abcd,w}^{\mathrm{FFT}}(+\omega_1,+\omega_2)+S_{abcd,w}^{\mathrm{FFT}}(+\omega_1,-\omega_2)\right| \tag{24}$$

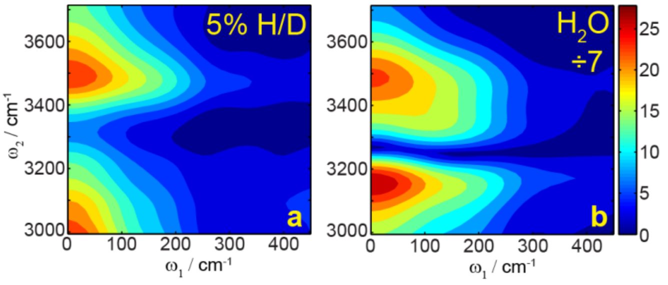

Supplementary Figure 8. Calculated 2D TIRV spectra for different isotopically diluted water. Absolute-value 2D TIRV spectra calculated by MD simulation for 5% H/D (**a**) and 100% H_2O (**b**) without convolution with the laser pulses.

To take into account the limited bandwidth of the laser pulses we convolute the calculated response function with the THz and IR laser pulses, which is given by:

$$\tilde{S}^{\mathrm{FFT}}_{abcd,w}(+\omega_1,\pm\omega_2) = S^{\mathrm{FFT}}_{abcd,w}(+\omega_1,\pm\omega_2)E_{THz}\left(\omega_1\right)E_{IR}\left(\omega_1\mp\omega_2\right), \quad (25)$$

where $E_{\mathrm{THz}}\left(\omega\right)$ and $E_{\mathrm{IR}}\left(\omega\right)$ are the spectra of the THz and IR laser pulses which were calculated as the square root of the corresponding experimentally measured intensity spectra(Fig. 2b,c, respectively). Because the $\omega_1 < 20\ \mathrm{cm}^{-1}$ spectral range in the experimental 2D TIRV spectra is not reliable, in the convolution we assume $E_{\mathrm{THz}}(\omega < 20\ \mathrm{cm}^{-1}) = 0$. The absolute-value 2D TIRV spectrum convoluted with the laser pulses (Fig. 3k,l) is given by:

$$I_{abcd}(\omega_1,\omega_2) = \left|\tilde{S}^{\mathrm{FFT}}_{abcd,w}(+\omega_1,+\omega_2) + \tilde{S}^{\mathrm{FFT}}_{abcd,w}(+\omega_1,-\omega_2)\right|. \quad (26)$$

Calculation procedure of the 2D TIRV *sin-sin* Fourier-transform spectra from simulated time domain signals.

The center frequency of the vibrational mode is not apparent from the 2D TIRV Fourier-transform spectra calculated by Supplementary Eq. (23) because the spectra contain the dispersive peak around the center frequency. Therefore, we use the *sin-sin* transformations to clearly reveal the center frequency of the vibrational mode from the absorptive peak. The frequency domain *sin-sin* Fourier transformed response function is defined as:

$$S^{\mathrm{SST}}_{abcd,w}(\omega_1,\omega_2) = \mathrm{Im}\left[\sum_{k=0}^{N_1-1}\exp\left[-\omega_1 k\Delta t_1\right]\mathrm{Im}\left[\sum_{l=0}^{N_2-1}\exp\left[-\omega_2 l\Delta t_2\right]S^{(3)}_{abcd,w}(k\Delta t_1, l\Delta t_2)\right]\right]. \quad (27)$$

Only the $\left(+\omega_1,+\omega_2\right)$ quadrant is calculated by Supplementary Eq. (27). Note that for the *sin-sin* Fourier transform we do not convolve the water response function with the laser fields.

Supplementary References

1. D’Angelo, F., Mics, Z., Bonn, M. & Turchinovich, D. Ultra-broadband THz time-domain spectroscopy of common polymers using THz air photonics. *Opt. Express* **22,** 12475 (2014).
2. Mukamel, S. *Principles of Nonlinear Optical Spectroscopy*. (Oxford University Press, 1995).
3. Shen, Y.-R. *The principles of nonlinear optics*. (Wiley, 1984).
4. Hasegawa, T. & Tanimura, Y. A polarizable water model for intramolecular and intermolecular vibrational spectroscopies. *J. Phys. Chem. B* **115,** 5545–53 (2011).
5. Tuckerman, M., Berne, B. J. & Martyna, G. J. Reversible multiple time scale molecular dynamics. *J. Chem. Phys.* **97,** 1990–2001 (1992).
6. Yoshida, H. Construction of higher order symplectic integrators. *Phys. Lett. A* **150,** 262–268 (1990).
7. Martyna, G. J., Klein, M. L. & Tuckerman, M. Nosé–Hoover chains: The canonical ensemble via continuous dynamics. *J. Chem. Phys.* **97,** 2635–2643 (1992).
8. Hasegawa, T. & Tanimura, Y. Nonequilibrium molecular dynamics simulations with a backward-forward trajectories sampling for multidimensional infrared spectroscopy of molecular vibrational modes. *J. Chem. Phys.* **128,** 64511 (2008).
9. Ito, H., Hasegawa, T. & Tanimura, Y. Calculating two-dimensional THz-Raman-THz and Raman-THz-THz signals for various molecular liquids: The samplers. *J. Chem. Phys.* **141,** 124503 (2014).
10. Mukamel, S., Khidekel, V. & Chernyak, V. Classical chaos and fluctuation-dissipation relations for nonlinear response. *Phys. Rev. E* **53,** R1–R4 (1996).
11. Jansen, T. l. C., Duppen, K. & Snijders, J. G. Close collisions in the two-dimensional Raman response of liquid carbon disulfide. *Phys. Rev. B* **67,** 134206 (2003).
12. Ito, H. & Tanimura, Y. Simulating two-dimensional infrared-Raman and Raman spectroscopies for intermolecular and intramolecular modes of liquid water. *J. Chem. Phys.* **144,** 74201 (2016).
